# Harmonization of Longitudinal Multi‐Scanner Neuroimaging Data With Nonlinear Biological Associations

**DOI:** 10.1002/hbm.70687

**Published:** 2026-11-13

**Authors:** Neel M. Desai, Haochang Shou, Noah Hillman, Randa Melhem, Lori L. Beason‐Held, Susan M. Resnick, Alzheimer's Disease Neuroimaging Initiative, Baltimore Longitudinal Study of Aging, Christos Davatzikos, Russell T. Shinohara

**Affiliations:** ^1^ The Penn Statistics in Imaging and Visualization Endeavor (PennSIVE), Center for Artificial Intelligence and Data Science for Integrated Diagnostics (AI2D), Department of Biostatistics, Epidemiology and Informatics University of Pennsylvania Philadelphia Pennsylvania USA; ^2^ Center for Biomedical Image Computing and Analytics University of Pennsylvania Philadelphia Pennsylvania USA; ^3^ Laboratory of Behavioral Neuroscience National Institute on Aging, National Institute of Health (NIH) USA; ^4^ Center for Biomedical Image Computing and Analytics, Department of Radiology University of Pennsylvania Philadelphia Pennsylvania USA

**Keywords:** ADNI–BLSA, Alzheimer's, ComBat, harmonization, MRI, semi‐parametric regression

## Abstract

The aggregation of neuroimaging data across multiple sources, typically scanners or imaging sites, has the potential to improve statistical power when conducting inference. The added benefits of increased sample size to inference, however, can be obstructed by unwanted source‐specific technical variability that increases difficulty in estimating biological signals of interest. To address this issue, recent methods based on ComBat, a batch‐correction procedure originally developed for genomics, have been developed for both cross‐sectional and longitudinal neuroimaging data. While these approaches have successfully removed technical batch‐specific variability, they restrict their consideration to linear biological associations. In this article we introduce a ComBat‐based method to harmonize longitudinal multisource imaging data that can account for nonlinear biological associations of interest. We demonstrate via simulation that, in the presence of nonlinear biological signal, our method outperforms existing ComBat‐based approaches designed for longitudinal imaging data, particularly in settings where observed values of relevant biological covariates lack substantial overlap across sources. Using volumetric measurements for 504 subjects combined from both the Baltimore Longitudinal Study of Aging (BLSA) and the Alzheimer's Disease Neuroimaging Initiative (ADNI), we identify the presence of both additive and multiplicative study‐specific effects for a substantial number of brain regions and demonstrate the effective removal of their presence on data harmonized by our method. The flexibility of the proposed method towards capturing many types of biological associations makes it a versatile procedure that can be used to harmonize longitudinal biomedical data.

## Graphical abstract

We develop a ComBat‐based approach to harmonize longitudinal multi‐source imaging data that can flexibly account for non‐linear biological associations of interest. Our method outperforms existing ComBat‐based harmonization approaches designed for longitudinal data.

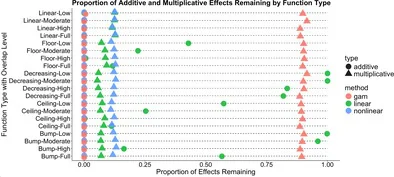

## 1 Introduction

In neuroimaging, harmonization methods seek to enable the effective aggregation of datasets across multiple sources; this strategy potentially increases statistical power for performing inference. These approaches attempt to preserve biological signals of interest while removing unwanted technical variability that occurs due to differences across scanner hardware, software, and site‐specific data acquisition strategies (Han et al. 2006; Jovicich et al. 2006; Takao et al. 2011). In recent years, a variety of harmonization approaches for neuroimaging data have been developed based on ComBat, an empirical Bayesian method originally designed for applications in genomics (Johnson et al. 2007). These approaches have been developed for diffusion tensor imaging (DTI) and radiomic features derived from positron emission tomography (PET) scans (Fortin et al. 2017, 2018; Yu et al. 2018; Orlhac et al. 2018). For cross‐sectional studies, a ComBat‐based harmonization method has been developed to assess structural brain changes across lifespan using generalized additive models that allow flexible biological associations (Pomponio et al. 2020). In a longitudinal context, a recent approach introduced a ComBat‐based framework for neuroimaging data that accounted for within subject‐correlation inherent to longitudinal datasets (Beer et al. 2020). This approach addressed limitations encountered in prior work seeking to harmonize longitudinal neuroimaging data (Venkatraman et al. 2015; Müller et al. 2016).

ComBat‐based harmonization enjoys several distinct advantages over other attempts at batch (i.e., site, scanner, or study) correction for aggregated neuroimaging data. First, the method is flexible to study design and only requires the same features to be measured for each participant observed. This property is distinct from other approaches effectively employed for harmonization that rely on overlap of participants between sites or scanners considered (Erus et al. 2018; Dewey et al. 2019). Second, ComBat‐based approaches have shown robustness to outliers. This property is due to the empirical Bayes framework employed by ComBat in contrast to approaches that treat batch as a fixed covariate (Johnson et al. 2007). Within each batch (i.e., site, scanner, or study), ComBat assumes that all feature‐specific batch effects derive from a common distribution, which allows information to be borrowed across features within a batch and shrunk towards a common mean. Furthermore, ComBat also removes heteroscedasticity of model errors across batch; this location‐scale adjustment has previously been shown to have benefits over approaches that solely adjust for mean effects (Fortin et al. 2018).

Inherent challenges exist when attempting to harmonize longitudinal neuroimaging data while preserving biological signal that has complex, potentially nonlinear biological associations. For longitudinal data, using cross‐sectional harmonization approaches ignores dependencies of within‐subject observations across time and can lead to the introduction of spurious biological signal due to inconsistencies in batch correction across time points. In addition, leveraging subject‐specific dependencies via a longitudinal method can lead to gains in statistical power (Beer et al. 2020). However, the presence of nonlinear signal can negate any potential benefits due to the introduction of spurious artifacts (Pomponio et al. 2020). The high risk of mischaracterizing biological associations highlights the importance of a method that is flexible enough to both capture complex biological associations and leverage additional structure inherent to longitudinal data.

In light of the challenges above, in this article we propose a method to harmonize multi‐scanner longitudinal neuroimaging data that flexibly preserves nonlinear biological associations. Our method accounts for within‐subject dependence inherent to longitudinal studies. In simulations designed with a variety of nonlinear biological signals, we demonstrate our approach's superior performance at removing batch‐specific technical effects over existing longitudinal ComBat‐based approaches; this improvement is most prominent in settings where biological covariates lack substantial overlap in observed values across batches. We demonstrate our approach using longitudinal volumetric data combined from the Alzheimer's Disease Neuroimaging Initiative (ADNI) (Weiner et al. 2015) and the Baltimore Longitudinal Study of Aging (BLSA) (Ferrucci 2008).

The rest of this article is organized as follows. Section 2 outlines our method in detail. Section 3 is a simulation study. Section 4 describes the combined ADNI–BLSA dataset, demonstrates the presence of study‐specific technical effects, and applies our approach to the ADNI–BLSA data. We conclude the article with a discussion in Section 5. Software is available for our approach at https://github.com/nmd1994/NonlinearLongitudinalComBat/.

## 2 Nonlinear Longitudinal ComBat

*Nonlinear longitudinal ComBat model*: We begin this section by outlining our overall proposed model. Given batch (i.e., scanner, site, study) index $i\in \left[1,\ldots ,m\right]$, participant index $j\in \left[1,\ldots ,N\right]$, participant‐specific time $t\in \left[1,\ldots ,K_{j}\right]$ (where *t* is not necessarily identical across subjects), covariate index $p\in \left[1,\ldots ,P\right]$, and feature v:

(1) $$y_{\mathrm{ijv}}\left(t\right)=\alpha_{v}+\gamma_{\mathrm{iv}}+\sum_{p=1}^{P}\;f_{\mathrm{vp}}\left(X_{\mathrm{jp}}\left(t\right)\right)+\eta_{\mathrm{jv}}+\delta_{\mathrm{iv}}\epsilon_{\mathrm{ijv}}\left(t\right),$$

where $y_{\mathrm{ijv}}\left(t\right)$ is the observed data for feature v, participant j, batch i, and time *t*, $\alpha_{v}$ is the overall mean for feature v at baseline, $\gamma_{\mathrm{iv}}$ is batch *i*'s additive effect for feature v, $X_{\mathrm{jp}}\left(t\right)$ is participant *j*'s possibly time‐varying covariate p that varies with $y_{\mathrm{ijv}}\left(t\right)$ by smooth, possibly nonlinear function $f_{\mathrm{vp}}$, $\eta_{\mathrm{jv}}$ is a subject‐specific random intercept for participant j and feature v, $\delta_{\mathrm{iv}}$ is batch *i*'s multiplicative scaling factor for feature v, and $\epsilon_{\mathrm{ijv}}\left(t\right)$ is an error term. $X_{\mathrm{jp}}\left(t\right)$ includes both baseline covariates that do not change with time (e.g., sex, baseline diagnosis) and time‐varying covariates such as age at visit time t. We assume the random intercepts $\eta_{\mathrm{jv}}$'s and error terms $\epsilon_{\mathrm{ijv}}\left(t\right)$'s are mutually independent and are distributed as $\eta_{\mathrm{jv}}∼N\left(0,\rho_{v}^{2}\right),\;\epsilon_{\mathrm{ijv}}\left(t\right)∼N\left(0,\sigma_{v}^{2}\right)$.

*Representing nonlinear fits as random effects*: To represent each function $f_{\mathrm{vp}}$ we utilize smoothing splines; smoothing splines use a spline basis with penalized coefficients to encourage smoothness to balance between interpolating observed trends and fitting each $f_{\mathrm{vp}}$ with a linear function. In particular, we use a parameterization of these penalized splines that separates linear and nonlinear components of each $f_{\mathrm{vp}}$ and enables each function's degree of nonlinearity to be estimated as variance components of random effects in a linear mixed model. Mathematical details on this parameterization can be seen in Wood (2017). Given this representation of penalized splines we observe:
(2)

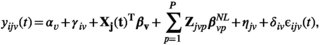

where $X_{j}{\left(t\right)}^{T}$ is a p×1 vector of potentially time‐varying covariates for participant j at time t, $\beta_{v}$ is a p×1 vector of coefficients for p covariates, $Z_{\mathrm{jvp}}$ is a 1×k vector of k nonlinear basis components of $f_{\mathrm{vp}}$ (i.e., the nonlinear portion of the basis representation for covariate p) for subject j, and $\beta_{\mathrm{vp}}^{\mathrm{NL}}∼N\;\left(0,\frac{I_{k}}{\lambda_{p}}\right)$ are the k×1 vector of coefficients for nonlinear basis functions $Z_{\mathrm{jvp}}$. Before providing explicit details on how we conduct harmonization, we represent our overall harmonized data in terms of our proposed model below:

$$\begin{array}{cc} y_{\mathrm{ijv}}^{\text{ComBat}}\left(t\right)=\; & \frac{y_{\mathrm{ijv}}\left(t\right)-{\hat{\alpha }}_{v}-{\hat{\gamma }}_{\mathrm{iv}}-X_{j}{\left(t\right)}^{T}{\hat{\beta }}_{v}-\sum_{p=1}^{P}Z_{\mathrm{jvp}}{\hat{\beta }}_{\mathrm{vp}}^{\mathrm{NL}}-{\hat{\eta }}_{\mathrm{jv}}}{{\hat{\delta }}_{\mathrm{iv}}}\\ & +{\hat{\alpha }}_{v}+X_{j}{\left(t\right)}^{T}{\hat{\beta }}_{v}+\sum_{p=1}^{P}Z_{\mathrm{jvp}}{\hat{\beta }}_{\mathrm{vp}}^{\mathrm{NL}}+{\hat{\eta }}_{\mathrm{jv}} \end{array}$$

### 2.1 Parameter Estimation

*Standardization step*: Before applying the empirical Bayes estimation of location‐scale parameters inherent to ComBat‐based harmonization, we first standardize features to have overall mean and variance on approximately the same scale to prevent biased estimates of batch effects (Johnson et al. 2007). In the context of our approach, we estimate fixed effect parameters ($\alpha_{v}$, $\beta_{v}$, $\gamma_{\mathrm{iv}}$) with the best linear unbiased estimator (BLUE) and produce unbiased estimates of variance components ($\rho_{v}^{2}$, $\sigma_{v}^{2}$, $\frac{I_{k}}{\lambda }$) with the restricted maximum likelihood (REML) estimator. Likewise, subject‐specific intercepts $\eta_{\mathrm{jv}}$ are estimated with the best linear unbiased predictor (BLUP). Standard constraints are imposed to ensure identifiability of nonlinear smooth components and random intercepts (Wood 2017). Standardized data is computed as follows:

$$z_{\mathrm{ijv}}\left(t\right)=\frac{y_{\mathrm{ijv}}\left(t\right)-{\hat{\alpha }}_{v}-X_{j}^{T}\left(t\right){\hat{\beta }}_{v}-\sum_{p=1}^{P}Z_{\mathrm{jvp}}{\hat{\beta }}_{\mathrm{vp}}^{\mathrm{NL}}-{\hat{\eta }}_{\mathrm{jv}}}{{\hat{\sigma }}_{v}}$$

We estimate, but do not remove, scanner additive effect $\gamma_{\mathrm{iv}}$ during the standardization step. Post‐standardization, we assume that $z_{\mathrm{ijv}}∼N\left(\gamma_{\mathrm{iv}},\delta_{\mathrm{iv}}^{2}\right)$. Batch‐specific effects are assumed to follow the following prior distributions: $\gamma_{\mathrm{iv}}∼N\left(\gamma_{i},\tau_{i}^{2}\right)$ and $\delta_{\mathrm{iv}}^{2}∼\mathrm{IG}\left(\omega_{i},\theta_{i}\right)$.

*Empirical Bayes estimation of batch effects*: Following standardization, additive and multiplicative batch effects are estimated by an empirical Bayes procedure typical of ComBat‐based approaches. From standardized data, method‐of‐moments (MoM) estimators are used to estimate hyperparameters ($\gamma_{i},\tau_{i}^{2},\omega_{i},\theta_{i}$) and ($\gamma_{\mathrm{iv}}$, $\delta_{\mathrm{iv}}^{2}$) are estimated by their conditional posterior means. Derivations for these empirical Bayes estimates can be seen in Johnson et al. (2007).

*Producing nonlinear longitudinal ComBat‐harmonized data*: Given additive and multiplicative batch effects estimated by empirical Bayes, ${\hat{\gamma }}_{\mathrm{iv}}$ and ${\hat{\delta }}_{\mathrm{iv}}$, we now provide an equation for data harmonized by our procedure:

(3) $$y_{\mathrm{ijv}}^{\text{ComBat}}\left(t\right)=\frac{{\hat{\sigma }}_{v}}{{\hat{\delta }}_{\mathrm{iv}}}\left[z_{\mathrm{ijv}}\left(t\right)-{\hat{\gamma }}_{\mathrm{iv}}\right]+{\hat{\alpha }}_{v}+X_{j}^{T}{\hat{\beta }}_{v}+\sum_{p=1}^{P}Z_{\mathrm{jvp}}{\hat{\beta }}_{\mathrm{vp}}^{\mathrm{NL}}+{\hat{\eta }}_{\mathrm{jv}}$$

To remove batch effects, a location‐scale adjustment is first performed to standardize data. Following this step, which puts features back on its original scale, the estimated biological variability of interest is then added back to the data.

We conduct all analysis using the R programming language. We fit all additive mixed models using the mgcv package (Wood and Wood 2015). To test for additive batch effects, we compared models fit with and without batch as a fixed effect using the package pbkrtest to carry out hypothesis tests with the Kenward–Roger approach. We tested for the presence of multiplicative batch effects using the non‐parametric Fligner–Killeen test on the residuals of our data.

## 3 Simulation Study

Given a description of our approach, we now design a simulation to demonstrate our method's benefits in the presence of longitudinal data with nonlinear biological signal. Specifically, we test our approach's performance against LongCombat and ComBat‐GAM. LongCombat is a harmonization method designed to correct batch effects for longitudinal data in the presence of linear biological signal and ComBat‐GAM is a cross‐sectional harmonization method designed to correct batch effects in the presence of nonlinear biological signal (Beer et al. 2020; Pomponio et al. 2020). We simulate 100 datasets per setting and vary the following between settings: functional form of biological signal, level of covariate overlap (i.e., range observed) between batches. We fix the number of batches to 2 per simulated dataset (of equal sample size), the number of features to 20 per dataset, the covariate considered (Age), and the number of subjects to 200 (each with observations at 5 time points). We simulate features from the normal distribution, generate batch‐specific additive and multiplicative noise from the normal and inverse‐gamma distribution, respectively, and can represent our simulated data from the following form: $y_{\mathrm{iv}}=f\left(\;{\mathrm{Age}}_{i}\right)+\epsilon_{i}$, where $v\;\epsilon \;\left[1,\ldots ,20\right]$ and $i\;\epsilon \;\left[1,\ldots ,1000\right]$. Simulated ages were randomly sampled and ranged from 10 to 90. Age overlap intervals are defined as follows: Low: $\;\text{Batch}\;1\;\epsilon \;\left[10,35\right]$–$\;\text{Batch}\;2\;\epsilon \;\left[30,90\right]$, Moderate: $\;\text{Batch}\;1\;\epsilon \;\left[10,40\right]$–$\;\text{Batch}\;2\;\epsilon \;\left[30,90\right]$, High: $\;\text{Batch}\;1\;\epsilon \;\left[10,60\right]$–$\;\text{Batch}\;2\;\epsilon \;\left[30,90\right]$, Full: $\;\text{Batch}\;1\;\epsilon \;\left[10,90\right]$–$\;\text{Batch}\;2\;\epsilon \;\left[10,90\right]$. Functional forms considered include the following: linear ($f\left(x\right)=\mathrm{X\beta}$), decreasing ($f\left(x\right)=-x^{3}$), floor (concave) ($f\left(x\right)=-\ln\left(x\right)$), ceiling ($f\left(x\right)=\ln\left(x\right)$), and bump ($f\left(x\right)=\text{logNormal}\left(x\right)$). We flag batch remaining batch effects if tests for their significance fall below a *p* value of 0.05 (with a Bonferroni correction applied). For results taken from our approach, we set the number of knots for each spline basis to k=45. Specific details about our simulations can be seen in our software, which can be found at https://github.com/nmd1994/NonlinearLongitudinalComBat/.

Simulation results, visualized in Figure 1, indicate that our method outperforms both LongCombat and ComBat‐GAM in removing additive effects when nonlinear biological signal is present. Importantly, our method also performs extremely similarly to LongCombat when biological signal is linear, providing evidence that our method is not meaningfully penalized for being overly flexible when our model is mispecified to the data. Linear longitudinal harmonization approaches, while effectively removing additive batch effects when signals are linear, do not remove these effects well when biological signal follows a nonlinear function. For removing multiplicative effects, our method outperformed ComBat‐GAM and performed comparably to LongCombat, which appeared to remove slightly more multiplicative batch effects when true biological signal was nonlinear. It is important to note that LongCombat can only preserve linear biological signals, meaning some signal beyond linear complexity will be lost during the removal of batch effects. The ability to remove multiplicative batch effects appears to slightly worsen when the overlap between the range of observed ages between batches is not substantial. For additive batch effects, the relationship between covariate overlap across batches and the ability to remove technical effects seems to be function‐specific. We note that our approach appeared to remove all additive batch effects and the majority of multiplicative effects regardless of the signal function considered or the level of overlap between observed covariates in each batch. An additional simulation setting in Table A1 of Appendix A shows similar performance for our method when an alternative smoother is used post‐harmonization, demonstrating robustness to smoother choice.

**FIGURE 1:**
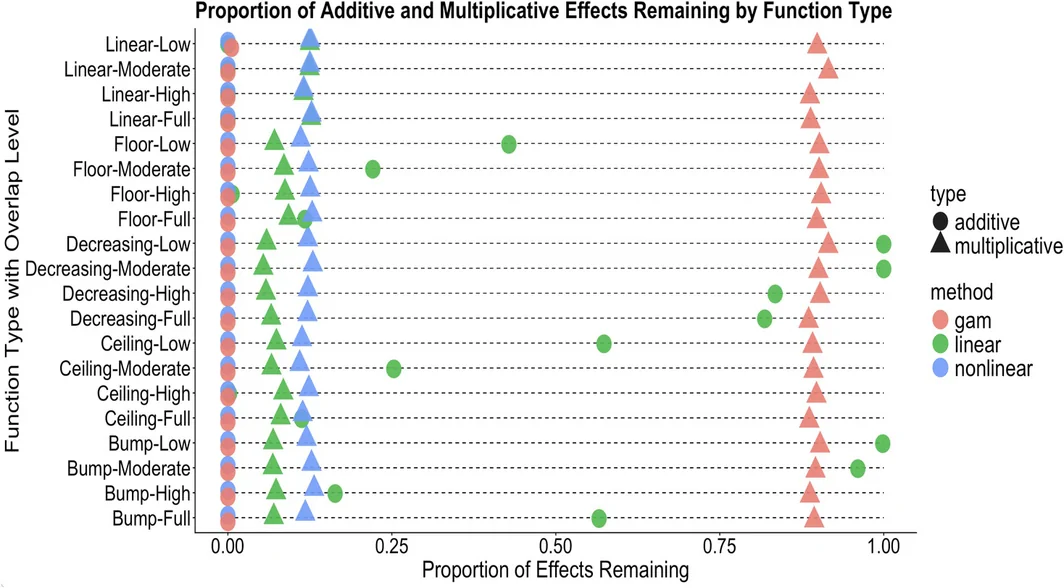
Figure visualizing simulation results. One hundred datasets were simulated per setting with the number of subjects fixed to 200 per setting, with observations at 5 time points for each subject. Twenty features were simulated for each dataset and two batches were generated for each dataset. Each dataset had additive and multiplicative batch effects simulated from a normal distribution. Each row represents simulations from a setting, which has a unique combination of functional form (bump, ceiling, decreasing, floor, linear) and level of overlap (low, moderate, high, full). For each setting, the proportion of additive and multiplicative batch effects remaining are shown (flagged as significant if below 0.05 *p* value with a Bonferroni correction). Triangles represent multiplicative batch effects and circles represent additive effects, while green represents results using LongCombat, red represents results from cross‐sectional ComBat‐GAM, and blue represents results from our nonlinear method.

*Null case: Method performance when no batch effects present*: We now examine the performance of our approach, ComBat‐GAM, and LongCombat under a variety of biological signals when *no* batch effects are present. To do this, we use the same functional forms for biological signal as our main simulation and restrict consideration to the Full overlap case (Full: $\;\text{Batch}\;1\;\epsilon \;\left[10,90\right]$–$\;\text{Batch}\;2\;\epsilon \;\left[10,90\right]$). We still simulate 100 datasets per setting with the number of subjects fixed to 200 per setting, with observations at 5 time points for each subject. Twenty features were simulated for each dataset and two batches were generated for each dataset. Our results, highlighted in Figure 2 below, show that our approach effectively avoids introducing spurious additive or multiplicative batch effects. Most notably, LongCombat appears to introduce substantial spurious additive batch effects when underlying biological signal is nonlinear, highlighting the importance of having a harmonization approach flexible enough to properly specify the shape of biological signal.

**FIGURE 2:**
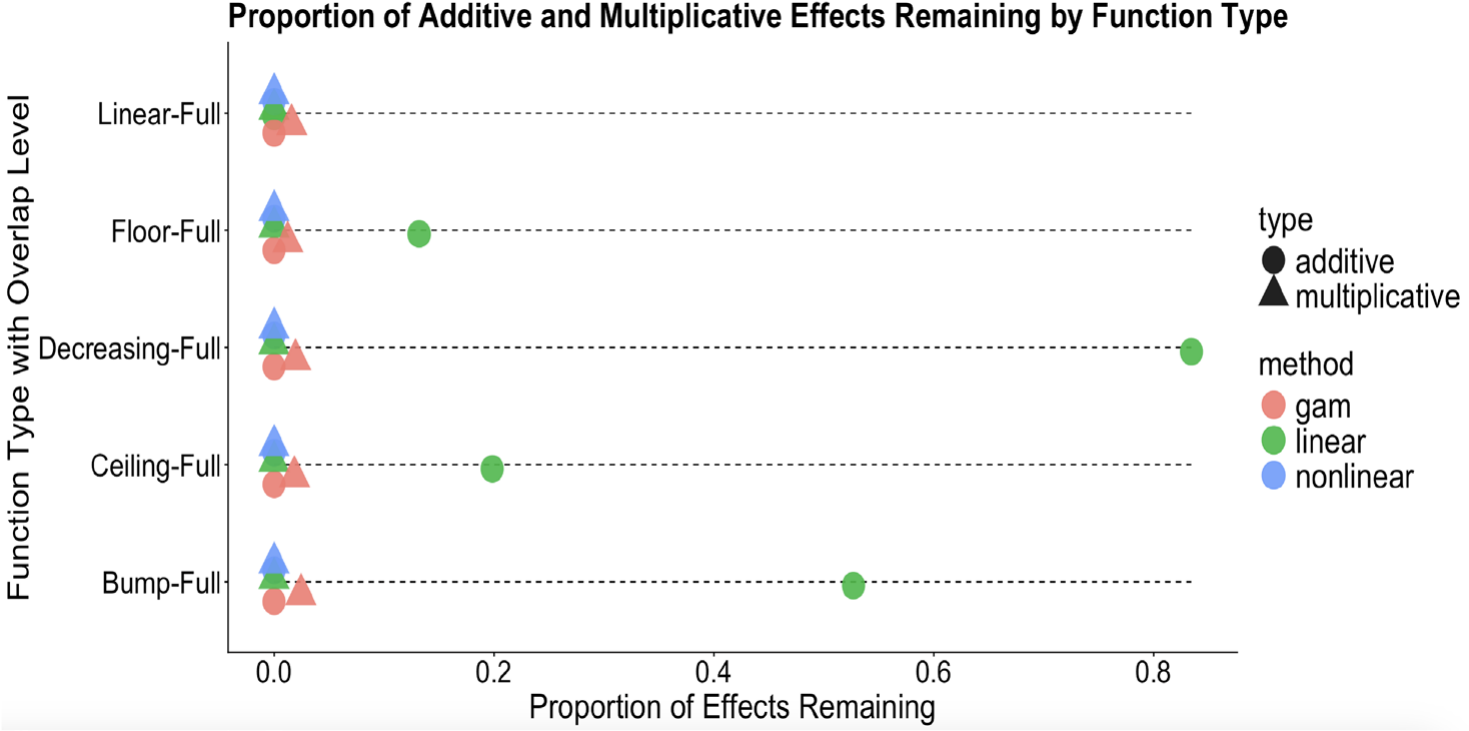
Figure visualizing simulation results. One hundred datasets were simulated per setting with the number of subjects fixed to 200 per setting, with observations at 5 time points for each subject. Twenty features were simulated for each dataset and two batches were generated for each dataset. Each dataset had no additive or multiplicative batch effects. Each row represents simulations from a setting, which has a unique functional form (bump, ceiling, decreasing, floor, linear). For each setting, the proportion of additive and multiplicative batch effects remaining are shown (flagged as significant if below a 0.05 *p* value with a Bonferroni correction). Triangles represent multiplicative batch effects and circles represent additive effects, while green represents results using LongCombat, red represents results from cross‐sectional ComBat‐GAM, and blue represents results from our nonlinear method.

## 4 Testing and Correcting for Batch Effects in Combined BLSA/ADNI Dataset

We analyzed longitudinal volumetric measurements for 504 subjects combined from both the BLSA (254 subjects) and the ADNI (250 subjects) (Weiner et al. 2015; Ferrucci 2008). ADNI acquired data from modalities including structural, functional, and molecular neuroimaging for participants across multiple sites using standardized protocols to maintain consistency during acquisition. Protocols provide guidelines for factors including imaging sequence parameters, scan timings and frequencies, data sharing, and other quality control procedures. For more detailed information on standardized protocols, including guidelines on scanner calibration and patient positioning, please refer to (https://adni.loni.usc.edu/). In contrast, the BLSA study follows cognitively healthy adults and follows them longitudinally for decades. The study acquires data via dedicated scanners to control for continuity from different patients across decades (Ferrucci 2008). For more information about BLSA, please refer to (https://www.blsa.nih.gov/). Ages for subjects from the BLSA study range from 27 to 96 while ages from ADNI ranged from 55 to 96 years old. We selected the following 20 anatomical areas based on visualizations indicating nonlinear associations with age: left accumbens area, right amygdala, left cerebellum white matter, right hippocampus, left pallidum, right putamen, cerebellar vermal lobules, left basal forebrain, left fornix, right anterior limb of internal capsule, left anterior orbital gyrus, right angular gyrus, left calcarine cortex, right central operculum, left posterior cingulate gyrus, right precuneus, left precentral gyrus, right planum temporale, left superior temporal gyrus, and right temporal pole. Along with the nonlinear function representing age, the following covariates were treated as additional linear fixed effects when performing this analysis: intracranial volume (DLICV) at baseline, sex, and diagnosis.

*Identifying and correcting batch effects*: Figure 3 shows a distribution of age in each study and Figure 4 shows, for a representative subset of ROIs, estimated age‐volume curves (adjusted for sex, intracranial volume (DLICV) at baseline, and diagnosis) for unharmonized data from both BLSA and ADNI. The results provide evidence for the presence of technical variability across batches, as estimations in overlapping age intervals between studies show clear differences. Tests for additive batch effects using pbkrtest (with Kenward–Roger method) and a Bonferroni correction showed 13 out of 20 features to have batch effects. Likewise, tests for multiplicative effects on residuals using the Fligner–Killeen test and a Bonferroni correction revealed 13 out of 20 features showed statistically significant heteroscedasticity. Results from data harmonized by our method showed no statistically significant additive effects using the Kenward–Roger method and a Bonferroni correction. Similarly, only 1 multiplicative effect remained on data harmonized by our method using the Fligner–Killeen test and a Bonferroni correction. Residuals of our analysis model fit to data harmonized by our method in Figure 5 similarly demonstrate no obvious multiplicative differences between batches for any features.

**FIGURE 3:**
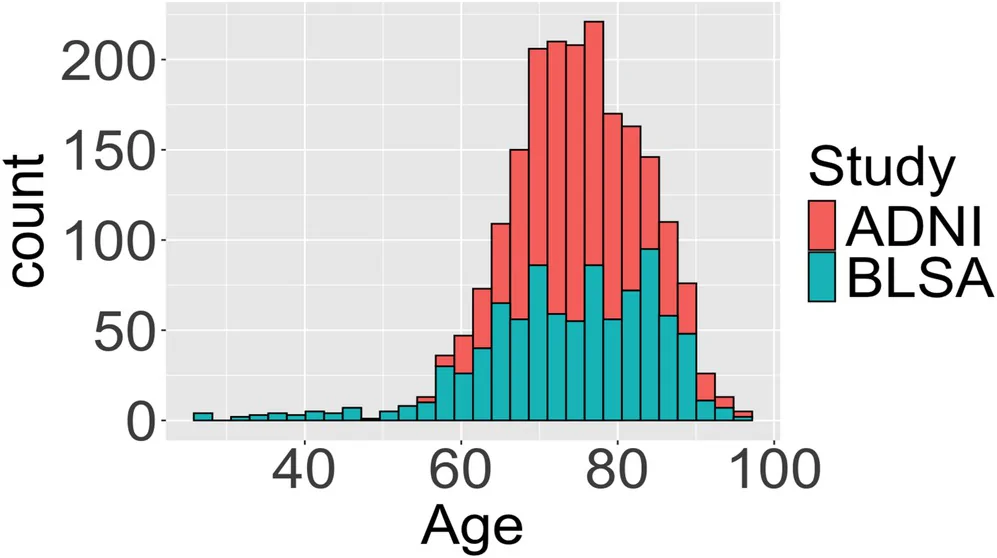
Histogram of ages for observations within the combined ADNI–BLSA dataset. Observations from the BLSA study are colored in blue while observations from the ADNI dataset are colored in red. Age ranges with data from both ADNI and BLSA have both colors overlapping, with their contribution to the range shown by the length of their color bar.

**FIGURE 4:**
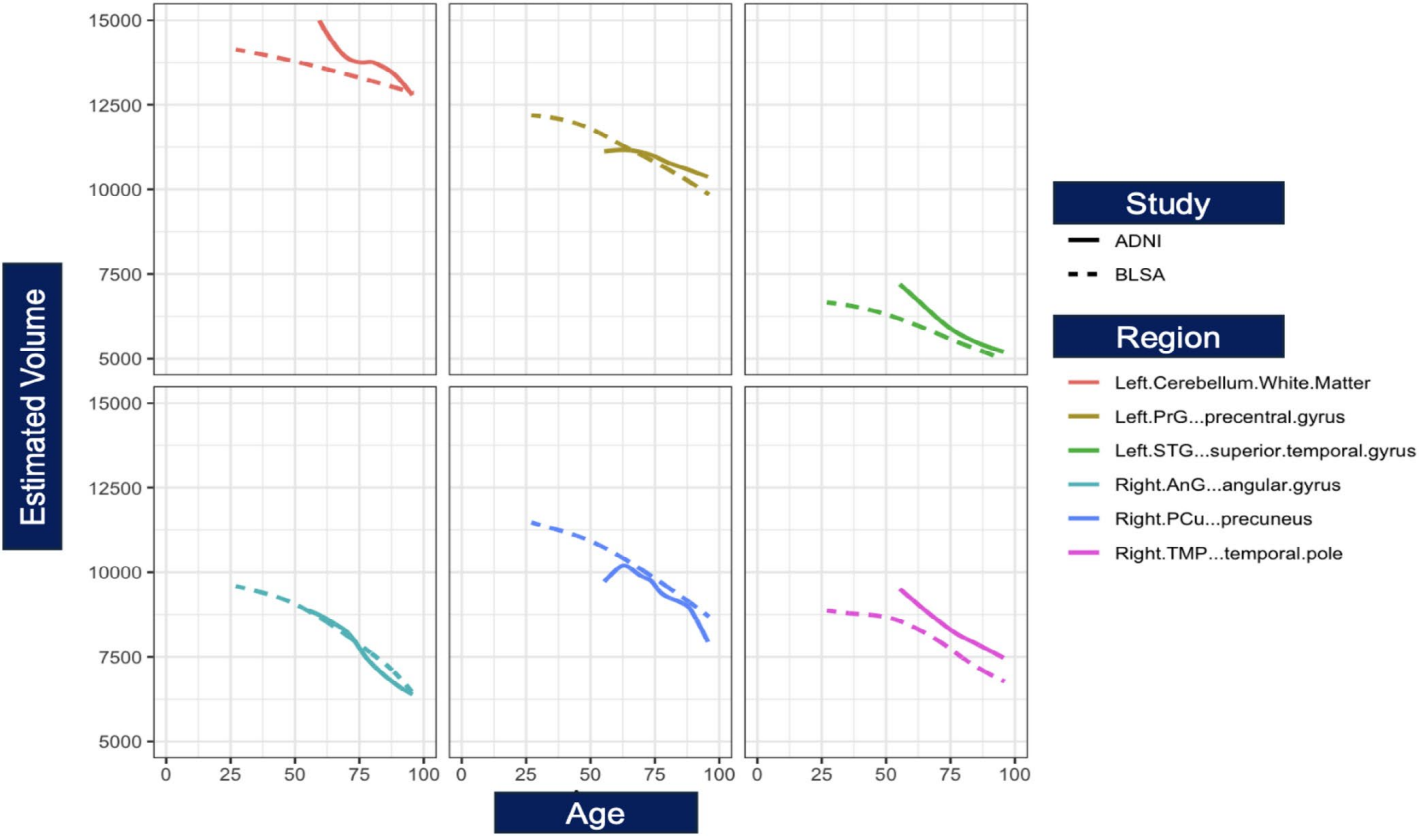
Age‐volume curves for a subset of ROIs estimated from our model (with DLICV, sex, and diagnosis as covariates) fit separately to unharmonized data from each of the ADNI and BLSA studies demonstrating gaps between fitted values at overlapping age intervals. Each ROI has a distinct color, estimated curves from BLSA are represented by dotted lines, and curves from ADNI are represented by solid lines. The estimated curves shown use average DLICV across patients, male, and clinically normal as the inputs for DLICV, sex, and diagnosis respectively.

**FIGURE 5:**
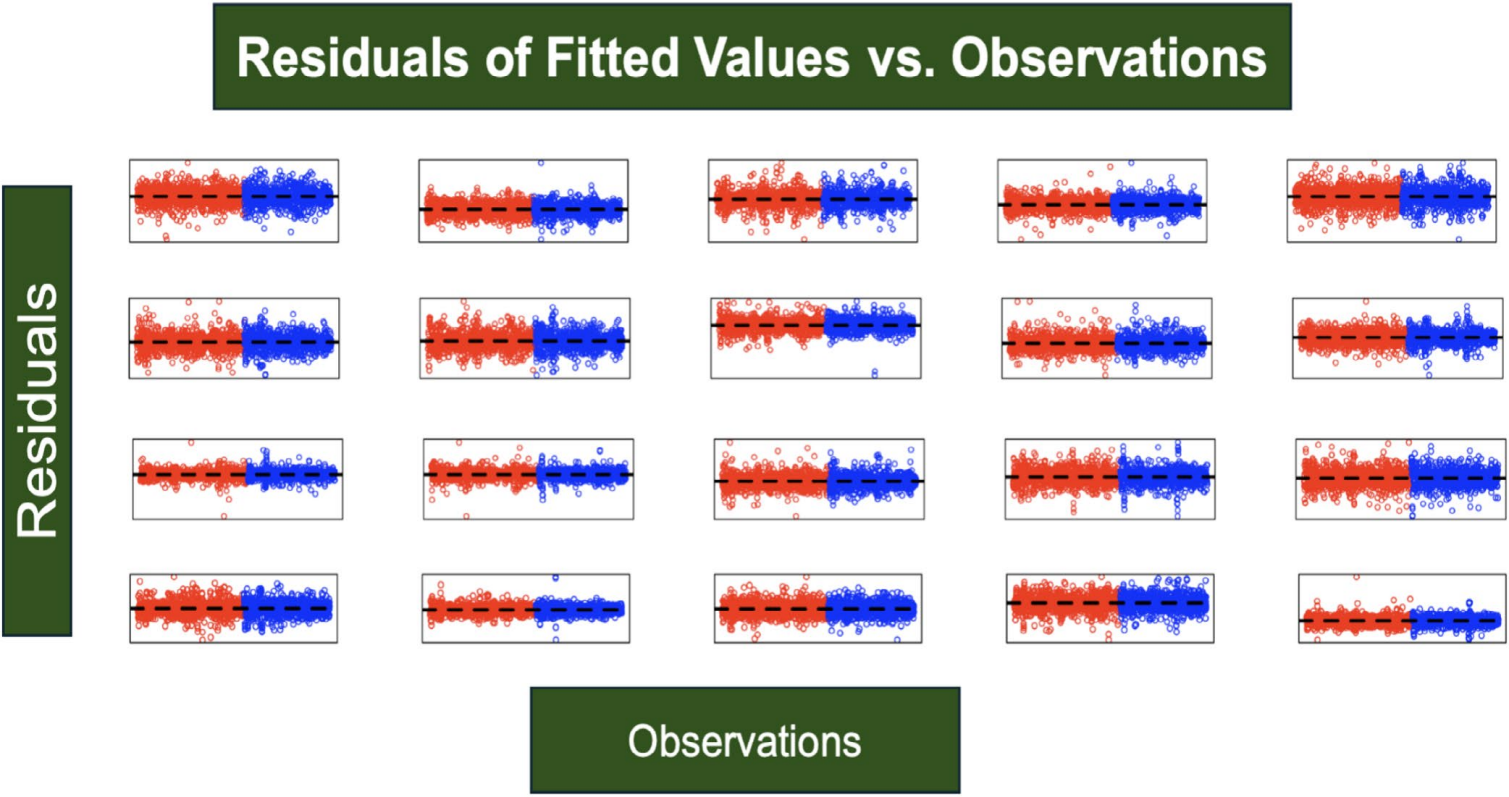
Residuals from our analysis model fit to data harmonized by our method. Each plot represents one of 20 regions of interest in our dataset. Observations are ordered by subject, with each subjects' visits ordered chronologically from first visit to last visit. The *y*‐axis shows the estimated residual for each observation while the *x*‐axis indexes each observation in the combined dataset. Blue dots represent observations from the ADNI study and red dots represent observations from the BLSA study.

As an additional validation step for our method, we also split our data into a training set and a test set to see how additive and multiplicative batch effects (estimated on the training set) performed at removing batch effects on the test set. We found that no additive batch effects and 2 multiplicative batch effects remained on the test set post harmonization, indicating very similar performance to our main analysis.

## 5 Discussion

In recent years, ComBat‐based approaches have been introduced as an effective solution for removing technical effects in neuroimaging data from multiple sources. These methods are flexible to study design and have been developed for both cross‐sectional neuroimaging data with flexible biological signal and for longitudinal neuroimaging data. In this article, we introduce a ComBat‐based method to harmonize longitudinal multisource imaging data with flexible, potentially nonlinear biological signals of interest. Through extensive simulations, we demonstrate the benefits of our method via its superior performance in the presence of nonlinear biological signal against both harmonization approaches for longitudinal data that restrict consideration to linear biological signal and against harmonization approaches that allow flexible nonlinear biological signal but are not designed to handle longitudinal data. We also show that our method performs very similarly to these approaches in the presence of linear biological signal, mitigating concerns about potential risks of added modeling flexibility. We also highlight the importance of properly specifying biological signal by showing that LongCombat introduces spurious additive batch effects in the null case (no batch effects present) when underlying biological signal is nonlinear. We apply our dataset to volumetric neuroimaging data from the ADNI and BLSA studies and show our method's effectiveness at removing additive and multiplicative batch effects, even in the presence of limited overlap in age ranges between studies (Ferrucci 2008; Weiner et al. 2015).

Our method has several limitations which present opportunities for future work. First, we assume biological signal that can be identified by a smooth, continuous function; some settings may not have signal that can be characterized by these types of shapes. Second, our method controls for longitudinal effects via a subject‐specific intercept; more complicated temporal structure may need to be addressed by a more intricate modeling framework. Finally, our method relies on technical variability to be identified by clear categorical differences such as scanner type, site, or study; future work could more comprehensively capture and correct technical variability in a way that is not directly addressed in this work. Examples of what a future approach could capture include differences in scanner vendor, differences in field strength, studies where participants switched scanners over time, and differences between studies in population selection (e.g., ADNI selected for cognitive impairment while BLSA selected for healthy volunteers). Future work could also evaluate the performance of our method on a broader set of metrics outside of strictly anatomically‐based ones.

Overall, we see our approach as a tool that expands the scope of ComBat‐based harmonization to include longitudinal data with complex biological patterns. The value of the proposed methodology may be especially important in real‐world data observed as part of clinical care longitudinally. We hope the added flexibility of our method, which should encompass a more general class of problems than existing approaches, allows researchers to effectively aggregate data and quantitatively address scientific questions that they were previously unable to answer.

## Funding

This work was supported by the National Institute of Mental Health (R01MH112847, R01MH123550, and R01MH12355), National Institute of Neurological Disorders and Stroke (R01NS112274 and U24NS13041), and National Institute on Aging (U01AG068057 and RF1AG054409).

## Data Availability

Data used in the preparation of this article were obtained from the ADNI database (adni.loni.usc.edu). The investigators within ADNI contributed to the design and implementation of ADNI and provided data but did not participate in analysis or writing of this report. A complete listing of ADNI investigators can be found at: https://adni.loni.usc.edu/wp‐content/uploads/how_to_apply/ADNI_Acknowledgement_List.pdf.

## Appendix A

*Sensitivity analysis (additional simulation setting testing robustness to alternative smoothers)*: We add an additional simulation setting performing validation post‐harmonization with a different smoother. Specifically, we ran a setting with the decreasing function, and performed validation post‐harmonization with a fixed spline set at k=45 knots. All other details of the simulation were kept the same as the main body of the manuscript. We note that each method performs similarly to the results in the main body of the manuscript when an alternative nonlinear model is used post‐harmonization to assess the proportion of batch effects remaining.

**TABLE A1:** Results showing the proportion of batch effects remaining (flagged as significant if below a 0.05 *p* value with a Bonferonni correction) for an additional simulation setting performing validation post‐harmonization with a fixed spline set at *k* = 45 knots.

	Additive	Multiplicative
Our method	0	0.12
LongCombat	0.89	0.07
ComBat‐GAM	0	0.90

*Note:* The underlying nonlinear function was set as decreasing and all simulation details were kept the same as those in the main body of the manuscript (i.e., 100 simulated datasets with both 200 subjects and 20 features each across 2 batches).
